# Community Characteristics and Leaf Stoichiometric Traits of Desert Ecosystems Regulated by Precipitation and Soil in an Arid Area of China

**DOI:** 10.3390/ijerph15010109

**Published:** 2018-01-10

**Authors:** Xiaolong Zhang, Tianyu Guan, Jihua Zhou, Wentao Cai, Nannan Gao, Hui Du, Lianhe Jiang, Liming Lai, Yuanrun Zheng

**Affiliations:** 1 Key Laboratory of Plant Resources, West China Subalpine Botanical Garden, Institute of Botany, Chinese Academy of Sciences, Xiangshan, Beijing 100093, China; zhangxiaolong@ibcas.ac.cn (X.Z.); guantianyu@ibcas.ac.cn (T.G.); zhoujihua@ibcas.ac.cn (J.Z.); caiwentao1990@hotmail.com (W.C.); gaonannan@ibcas.ac.cn (N.G.); duhui@ibcas.ac.cn (H.D.); jianglh@ibcas.ac.cn (L.J.); lailiming@ibcas.ac.cn (L.L.); 2 College of Resources and Environment, University of Chinese Academy of Sciences, Beijing 100049, China

**Keywords:** precipitation, community characteristics, leaf stoichiometric traits, soil properties, desert ecosystem, global climate change

## Abstract

Precipitation is a key environmental factor determining plant community structure and function. Knowledge of how community characteristics and leaf stoichiometric traits respond to variation in precipitation is crucial for assessing the effects of global changes on terrestrial ecosystems. In this study, we measured community characteristics, leaf stoichiometric traits, and soil properties along a precipitation gradient (35–209 mm) in a desert ecosystem of Northwest China to explore the drivers of these factors. With increasing precipitation, species richness, aboveground biomass, community coverage, foliage projective cover (FPC), and leaf area index (LAI) all significantly increased, while community height decreased. The hyperarid desert plants were characterized by lower leaf carbon (C) and nitrogen/phosphorus (N/P) levels, and stable N and P, and these parameters did not change significantly with precipitation. The growth of desert plants was limited more by N than P. Soil properties, rather than precipitation, were the main drivers of desert plant leaf stoichiometric traits, whereas precipitation made the biggest contribution to vegetation structure and function. These results test the importance of precipitation in regulating plant community structure and composition together with soil properties, and provide further insights into the adaptive strategy of communities at regional scale in response to global climate change.

## 1. Introduction

Water availability is the most important factor affecting ecosystem structure and function [1,2,3]. In arid and semi-arid regions, especially, biophysical activity is tightly coupled to water availability, and water deficit exerts a profound influence on ecosystem dynamics [4,5]. Precipitation is a key environmental factor that determines water availability in desert ecosystems and regulates the responses of plant communities and entire ecosystems [6,7,8]. The response and adaptation of desert plant community characteristics and their leaf stoichiometric traits to precipitation are of the utmost importance, as community characteristics and leaf traits are fundamental elements in understanding the structure and function of ecosystems [9,10,11]. Precipitation affects community characteristics and leaf traits in complex ways, especially in a changing climate. It may directly influence plant activity and ecological processes, and also indirectly mediates these through interactions with abiotic factors such as soil water content, supplementary water, soil nutrition, and evaporation [1,7,12,13]. Exactly how precipitation affects community characteristics and leaf stoichiometric traits across a precipitation gradient remains largely unclear and needs further research at the interface of ecology and hydrology, especially in arid regions [8].

Dryland ecosystems, which cover nearly 40% of terrestrial habitats and provide critical ecosystem services to biota, are particularly suitable for investigating the role of precipitation on ecological communities [2]. These ecosystems are defined by high seasonal and annual variation in precipitation [4]. In arid regions, sparse and variable precipitation exerts strong control over plant community composition, life histories, physiological properties, and resource availability, thereby impacting eco-hydrological processes [6,14]. The response of plants to precipitation regimes had been analyzed in some studies, within individual species, and within communities and ecosystems [1,14,15]. The research on the response of individual plants to precipitation regimes had focused mainly on biophysiological traits including water use efficiency, photosynthesis, sap flow, respiration and evapotranspiration [14,16,17]. Community characteristics including species diversity, species composition, and biomass are the main parameters of the ecological processes that are highly sensitive to precipitation [1,5,18,19]. Precipitation plays a crucial role in shaping vegetation distribution within arid environments, and understanding responses of community to precipitation is critical to maintain desert ecosystems sustainability [4,14].

Leaf traits are an important determinant of plant growth and production in plant communities, and their nutrient traits are closely associated with the structure and function of terrestrial ecosystems [9,10,20]. Elser et al. [21,22] revealed that terrestrial plants or plant communities grow in a wider range of nutrient conditions and the C/N/P stoichiometry of terrestrial plants could reflect how plant species adjusted to the local growth conditions. Ecological stoichiometry, as an integrative approach, can yield new insights for studying how precipitation affects the balance of essential elements including C, N, and P at different trophic levels and wide spatial scales [20,23,24,25]. Precipitation may change the leaf C, N, P stoichiometry through a cascade of plant-soil feedbacks, and may also alter the species composition and leaf C, N, P stoichiometry due to differences in species’ nutrition stoichiometry contents [13,26,27,28]. Currently, there has been increasing research on the leaf stoichiometry of plant species in relation to variations in geographical and climatic factors [20,25,26,28]. However, few studies have focused on the effects of precipitation regimes on the leaf stoichiometric traits of desert plants, especially the relationship between precipitation and nutrient stoichiometry in field environments across a range of precipitation gradients in arid regions. 

The middle and lower reaches of the Heihe River, a typical arid inland river desert ecosystem, occur in an extremely arid inland region where the ecological environment is fragile [29]. The primary landscapes are peripheral desert, riparian forest and a central oasis, with desert playing a crucial role in maintaining a stable ecological environment and the oasis in maintaining agricultural production. Over the past years, human activity (e.g., grazing and agriculture) and global climate change has led to the destruction of desert vegetation in this region [29,30,31,32]. Currently, some studies on vegetation-environment relationships have been carried out in several riparian and oasis zones [33,34,35,36]. However, little is known about the effects of precipitation on natural desert vegetation and soil properties at a regional scale, especially leaf stoichiometric traits. With a large range of annual precipitation from 29 to 447 mm, the middle and lower reaches of the Heihe River are a suitable study site for investigating the responses of natural desert ecosystems to precipitation.

Seven sites with natural desert vegetation in the middle and lower reaches of the Heihe River were used to explore patterns of community structure and leaf stoichiometric traits along a natural precipitation gradient. Specifically, the study aims to: (1) characterize the pattern of plant community characteristics, leaf stoichiometric traits and soil properties along precipitation gradient; (2) evaluate the relationship between plant community characteristics and soil properties, leaf stoichiometric traits and soil properties; and (3) determine major factors affecting community characteristics and leaf stoichiometric traits along a precipitation gradient. We hypothesized that precipitation play a major role on plant community structure, while soil properties had key influence on leaf stoichiometric traits. These findings can be useful to maintain the sustainability of natural desert ecosystems.

## 2. Materials and Methods 

### 2.1. Study Area and Site Description

**Figure 1:**
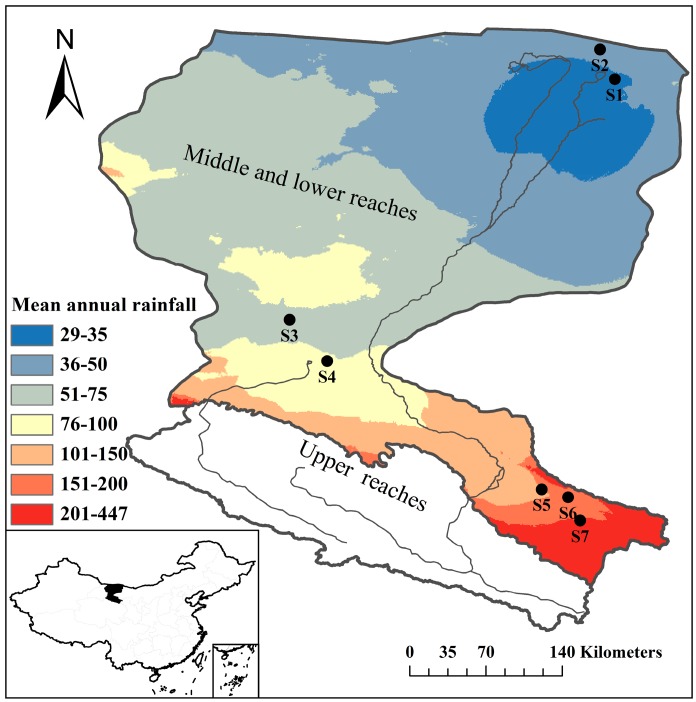
The Heihe River Basin in northwestern China and the locations of the sampling sites.

The Heihe River Basin (Figure 1) is the second largest inland river basin in Northwest China, with a length of 821 km in its main stream and a catchment area of 14.29 × 10^4^ km^2^. The river originates from the middle of the Qilian Mountains, on the northern Tibet Plateau, then flows through Qinghai Province, Gansu Province, and the Inner Mongolia Autonomous Region, and terminates at the north end of Juyan Lake in Ejin county, Inner-Mongolia [30]. The Heihe River Basin has a varied topography, with elevations between about 900 and 5500 m (calculated from ASTER GDEM, http://westdc.westgis.ac.cn/), and the integrated topographic landscape can be divided into: a glaciology and geocryology zone, an alpine vegetation zone, a piedmont oasis zone, and a desert zone. The desert zone accounts for more than 75% of the total land area. The upper reaches are covered with thick vegetation and have well-developed glaciology and geocryology, which means they form the main runoff generating region [37]. The middle and lower reaches have a great deal of farmland and desert, and have become the primary runoff consumption region [30].

The study was conducted in the vast natural desert, located in the middle and lower reaches of the Heihe River. The region is characterized by a typical continental arid climate, which is dominated by a warm-humid summer and a cold-dry winter. The mean annual precipitation (from 1950 to 2000) was 29–447 mm (calculated from the WORLDCLIM dataset, www.worldclim.org) with a high spatial and temporal variability. The majority (more than 75%) of precipitation falls from July to August, and pan evaporation is relatively high, especially in lower reaches, evaporation is 100 times greater than the precipitation [17,30]. The mean annual temperature is 5–10 °C. The soils have developed from gray-brown desert soil [30]. Natural desert vegetation accounts for 87.02% of the total area [38]. In the oasis, the primary vegetation is *Populus euphratica* Oliv. and *Tamarix ramosissima* Lebed. Outside of the oasis, the primary vegetation is temperate desert shrubland. Desert shrub plants (*Artemisia desertorum* Spreng., *Kalidium gracile* Fenzl, *Salsola passerina* Bunge, *Kalidium cuspidatum* (Ung. Sternb.) Grub., and *Haloxylon ammodendron* (C. A. Mey.) Bunge are the dominant species and major primary producers, and herb plant appeared in the form of companion species [30]. The list of plant species in the seven sampling sites were shown in Table A1.

### 2.2. Experimental Design and Data Collection

This study was conducted in temperate desert shrubland through the middle and lower reaches of Heihe River Basin. In August 2015, seven shrubland sampling sites along a precipitation gradient were prepared; vegetation and soil sampling were finished at the same time within one week when aboveground biomass reached the peak [30]. The sites were in an open, flat, undisturbed desert Gobi area, far from the river (more than 10 km) and other water resources. In each sampling site, three shrub quadrats (5 m × 5 m) were established randomly as three replicates. The number of species, coverage, plant height, leaf area index (LAI), basal diameter, and width of canopy were recorded individually. Four herb quadrats (1 m × 1 m) were established at each corner of the shrub quadrat to collect data of number of herb species, coverage, and height. Compared to community coverage, foliage projective cover only records green leaves coverage [39]. A 30 m sample lines were set up near each shrub quadrat, and foliage projective cover (FPC) was measured with a simple FPC measuring tube [39]. Aboveground biomass was determined by the harvest method. Harvested materials were oven-dried at 80 °C to constant weight, and then the weight was recorded. The geographic coordinates and elevation of each plot were recorded using a global positioning system. LAI was measured with a LAI-2200 Plant Canopy Analyzer (LI-COR, Lincoln, NE, USA), by using one sensor with a 90° view cap. Measurements were made near sunset [40].

At each site, intact soil cores were collected using a cutting ring (volume of 100 cm^3^) from five soil depths (0–10, 10–20, 20–30, 30–40, and 40–50 cm) in each shrub quadrat after removing any rocks and litter, with three replicates. Soil samples were sealed in an ice chest and were transported to laboratory. Soil samples were air-dried and passed through a 2-mm sieve. Soil pH and electrical conductivity (EC) were measured in 1:1 soil-water and 1:5 soil-water suspensions (Multiline F/SET-3, WTW, Weilheim, Germany), respectively [41]. Soil total C and N were measured using a C/H/N analyzer (Vario EL III, Elementar, Hanau, Germany) [42], soil available P was measured by the Olsen method, and soil available K was obtained with 1 M ammonium acetate and measured by atomic absorption spectroscopy [43]. Soil bulk density and gravimetric soil water content (SWC) was measured by collecting soil cores from each soil layer using a stainless-steel cutting ring (100 cm^3^) at 0–10, 10–20, 20–30, 30–40, and 40–50 cm depths in each shrub quadrat, and then were oven dried at 105°C to a constant weight.

### 2.3. Leaf Stoichiometric Traits 

Sun-exposed and fully expanded mature leaves (or assimilating shoots) were collected from three individuals of the dominant species at each shrub quadrat. The leaves from same species were put together and grouped in paper envelopes. Leaf stoichiometric traits were analyzed with three replicates for same sample, and presented in mass basis (%). The total C and N concentrations in the leaves were measured using a C/H/N analyzer (Vario EL III, Elementar, Hanau, Germany). Leaf P and K concentrations were measured using an inductively coupled plasma optical emission spectrometer (iCAP 6300, Thermo Scientific, Waltham, MA, USA) [44].

### 2.4. Statistical Analysis 

Species richness was determined from the total species numbers in each plot. Species importance value was calculated as (RD + RC + RF)/3 to indicate the dominant species (Table A2 and Table 1), where RD, RC, and RF are the relative density, relative coverage, and relative frequency, respectively, of each species in each plant community [45]. Gravimetric soil water content data were averaged across three soil layers of 0–10, 10–30, and 30–50 cm; other soil data were averaged across 0–50 cm soil depth.

All data were log 10 transformed to meet the homogeneity of variance and normality. One-way ANOVA analysis of variance was applied to compare the differences in community characteristics, leaf stoichiometric traits, and soil properties in different sites. If significant differences were found, Tukey’s test was used to determine the differences (Table A3 and Table A4). Regression analyses were used to detect relationship among plant community characteristics, leaf stoichiometric traits, and soil properties along precipitation gradient. Appropriate regression equations were selected based on level of significance and high R^2^ value. Pearson correlation was used to determine the strengths of possible relationships between community characteristics, leaf stoichiometric traits, and environmental factors. Significant differences were evaluated at the level of *p* < 0.05. Statistical analyses were carried out using SPSS Version 18.0 (SPSS, Chicago, IL, USA). 

Environment variables included precipitation and 11 soil properties including gravimetric soil water content (0–10 cm), gravimetric soil water content (10–30 cm), gravimetric soil water content (30–50 cm), soil bulk density, soil total nitrogen, soil total carbon, soil C/N, soil available P, soil available K, soil pH, and soil electrical conductivity were used to separate key environmental factors for variation of community characteristics, the marginal and conditional effects of the variables were calculated through forward selection in redundancy analysis (RDA) that directly showed the significance and percentage of the explained factors [46]. Statistical test for each added variable was conducted with Monte Carlo permutation tests (9999 permutations). Marginal effects showed the effects of the environmental variables on community characteristics, and conditional effects showed the effects of the environment variables on community characteristics after the anterior variable was eliminated by the forward selection method [31,32,46]. The forward selection method was performed to exclude variables that did not contribute significantly (*p* > 0.05) to variation, and the redundant variables were eliminated and a group of key variables was determined. Both precipitation variable and soil properties were included in the group of key variables, variation partitioning was used to separate the variation in the community characteristics between two groups of significant predictors: precipitation and soil properties. The independent effects of each factor and the interactive effects between factors were included in the final model [47]. Either precipitation variable or all of soil properties was not included in the group of key variables, variation partitioning procedure was not performed. Leaf stoichiometric traits data was analyzed in same process. The forward selection, Monte Carlo test, and variation partitioning were conducted using CANOCO for Windows program (version 5.0) [46].

## 3. Results

### 3.1. Changes in Community Characteristics along the Precipitation Gradient 

Species richness (*F* = 38.79, *p* < 0.001), aboveground biomass (*F* = 8.75, *p* < 0.001), community height (*F* = 18.51, *p* < 0.001), community coverage (*F* = 62.05, *p* < 0.001), FPC (*F* = 14.80, *p* < 0.001) and LAI (*F* = 76.44, *p* < 0.001) were significantly different among sites with different annual precipitation (Table 1 and Table A3). 

**Figure 2:**
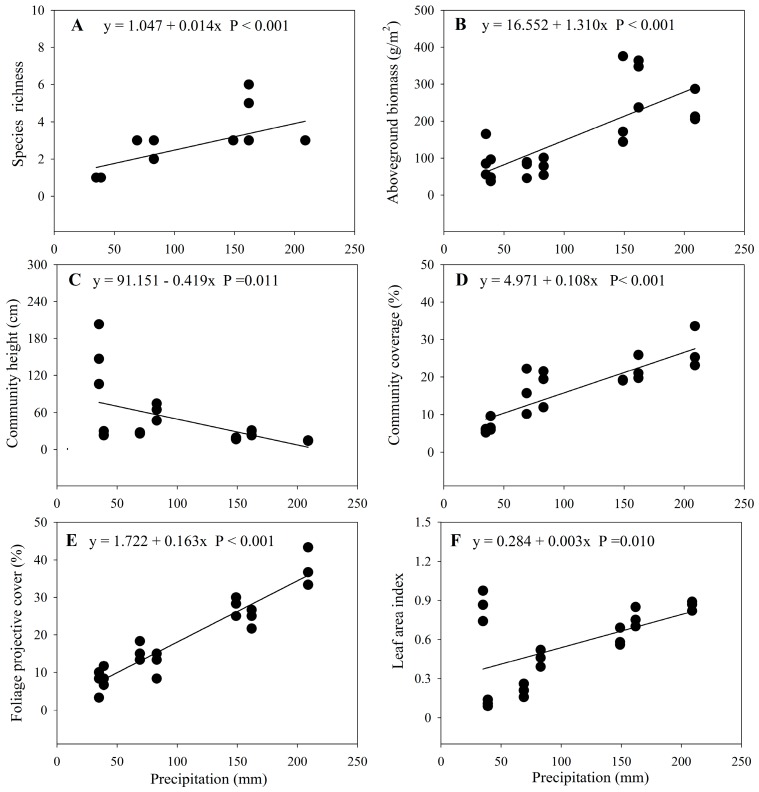
Changes of community characteristics with the precipitation gradient. (**A**) Species richness; (**B**) Aboveground biomass (g/m^2^); (**C**) Community height (cm); (**D**) Community coverage (%); (**E**) Foliage projective cover (%); (**F**) Leaf area index.

Species richness, aboveground biomass, community coverage, FPC, and LAI significantly increased with increasing precipitation and could be described by linear equations, while community height significantly decreased with increasing precipitation and could also be described by linear equations (Table 1, Figure 2). 

### 3.2. Changes in Leaf Stoichiometric Traits along the Precipitation Gradient

**Figure 3:**
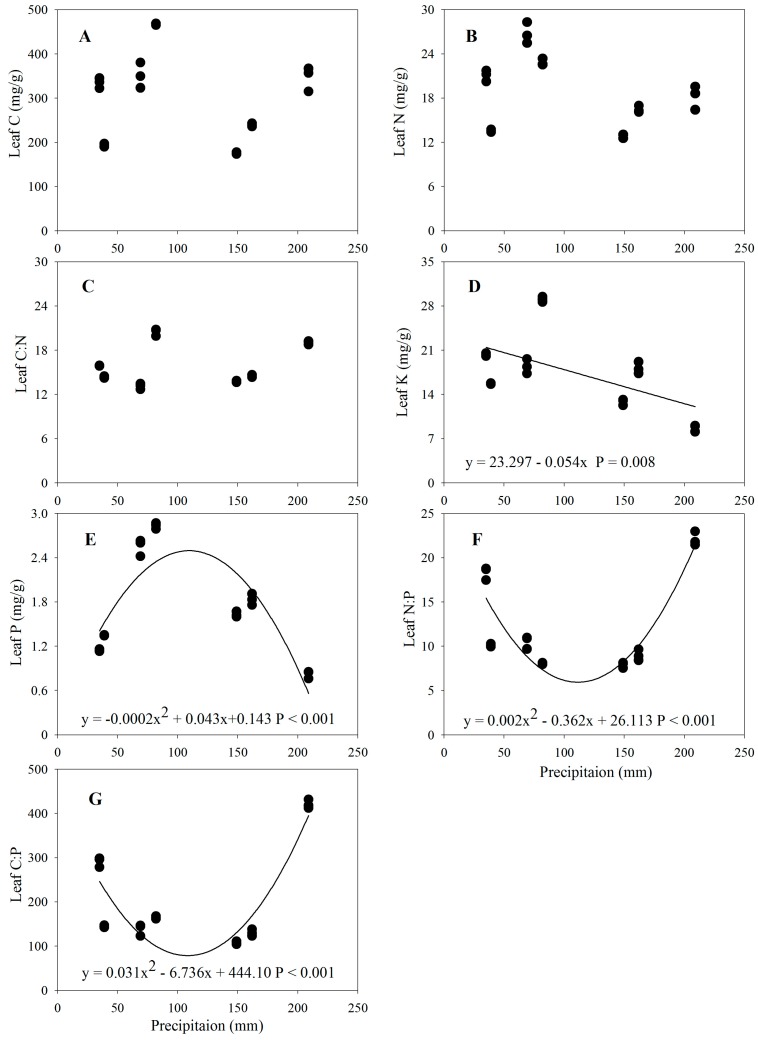
Changes of leaf C, N, P, K, and C/N/P ratios with the precipitation gradient. (**A**) Leaf C (mg/g); (**B**) Leaf N (mg/g); (**C**) Leaf C/N ratio; (**D**) Leaf K (mg/g); (**E**) Leaf P (mg/g); (**F**) Leaf N/P ratio; (**G**) Leaf C/P ratio; Lines are plotted if regressions are significant at *p* < 0.05.

For all species, the mean leaf C, N, P, and K contents, and C/N, C/P, and N/P ratios were 301.22 mg g^−1^, 18.81 mg g^−1^, 1.74 mg g^−1^, 17.59 mg g^−1^, 15.88, 199.68, and 12.27, respectively (Table 2). Leaf C (*F* = 175.76, *p* < 0.001), leaf N (*F* = 109.19, *p* < 0.001), leaf P (*F* = 456.43, *p* < 0.001), leaf K (*F* = 253.59, *p* < 0.001), leaf C/N (*F* = 319.26, *p* < 0.001), leaf C/P (*F* = 306.01, *p* < 0.001), and leaf N/P (*F* = 241.23, *p* < 0.001) were significantly different among sites with different precipitation (Table 2 and Table A3). Leaf C, leaf N, and leaf C/N had no significant trend with increasing precipitation. Leaf K decreased significantly with increasing precipitation and could be described by linear equation, while Leaf P showed a hump-shaped pattern, increasing and then decreasing rapidly with increasing precipitation, and could be described by quadratic curve (Figure 3). Leaf C/P and leaf N/P had the opposite trend to leaf P (Figure 3). 

### 3.3. Changes in Soil Properties along the Precipitation Gradient

Gravimetric soil water content in the 0–10 cm soil layer (GSWC10) (*F* = 3.24, *p* = 0.033), gravimetric soil water content in 10–30 cm soil layer (GSWC30) (*F* = 12.62, *p* < 0.001), gravimetric soil water content in 30–50 cm soil layer (GSWC50) (*F* = 18.01, *p* < 0.001), soil bulk density (*F* = 24.39, *p* < 0.001), soil total N (*F* = 22.26, *p* < 0.001), soil total C (*F* = 56.56, *p* < 0.001), soil C/N ratio (*F* = 28.68, *p* < 0.001), soil available P (*F* = 23.76, *p* < 0.001), soil available K (*F* = 24.02, *p* < 0.001), soil pH (*F* = 2.33, *p* = 0.41), and soil EC (*F* = 25.36, *p* < 0.001) varied significantly among sites with different precipitation (Table 3 and Table A4).

**Figure 4:**
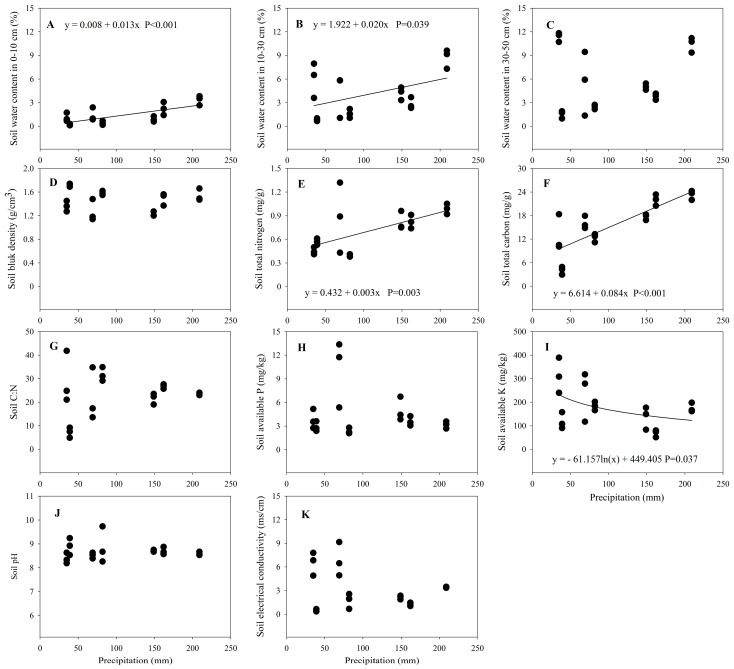
Changes of soil properties with the precipitation gradient. (**A**) Gravimetric soil water content in the 0–10 cm soil layer (%); (**B**) Gravimetric soil water content in 10–30 cm soil layer (%); (**C**) Gravimetric soil water content in 30–50 cm soil layer (%); (**D**) Soil bulk density (g/cm^3^); (**E**) Soil total N (mg/g); (**F**) Soil total C (mg/g); (**G**) Soil C/N ratio; (**H**) Soil available P (mg/kg); (**I**) Soil available K (mg/kg); (**J**) Soil pH; (**K**) Soil EC (ms/cm). Lines are plotted if regressions are significant at *p* < 0.05.

Gravimetric soil water content at 0–10 cm and at 10–30 cm showed a significantly increasing trend with increasing precipitation, and could be described by linear equations, but this trend was not significant at 30–50 cm soil depths (Figure 4). Soil bulk density and soil pH remained relatively constant with increasing precipitation (Figure 4). Soil total N and total C significantly increased with precipitation and could be described by linear equations, while soil available K significantly decreased with precipitation and could also be described by logarithmic equation (Figure 4). Soil C/N, soil available P, and soil electrical conductivity did not significantly vary with increasing precipitation (Figure 4). 

### 3.4. Relationships among Community Characteristics, Leaf Stoichiometric Traits and Environmental Factors

Species richness, aboveground biomass, community coverage, FPC, and LAI were significantly and positively related to soil water content at 0–10 cm. Species richness, FPC, and LAI was positively correlated with soil water content at 10–30 cm, and LAI was positively correlated with soil water content at 30–50 cm. There were other positive or negative correlations among community characteristics or leaf stoichiometric traits and soil properties in these natural desert communities (Table A5).

### 3.5. Controlling Factors of Community Characteristics and Leaf Stoichiometric Traits

**Figure 5:**
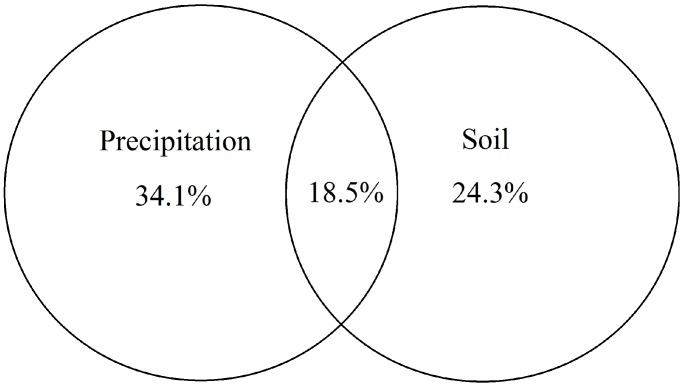
Variation partitioning of precipitation, soil properties, and their interactions in accounting for the variation of community characteristics. The numbers indicate the explanation percentage of variables and their interactions for variation.

In the Monte Carlo test of forward selection (*p* < 0.05) for community characteristics, precipitation, soil C/N, GSWC50, soil total nitrogen and soil available P passed the test (Table 4). Variation partitioning showed that precipitation and soil properties jointly explained 76.9% of the variation of community characteristics; precipitation had the largest contribution (34.1%), next was soil properties (24.3%), and then the interaction of precipitation and soil properties (Figure 5). In the Monte Carlo test of forward selection (*p* < 0.05) for leaf stoichiometric traits, GSWC50, soil C/N, soil bulk density, GSWC30, soil electrical conductivity and soil available K passed the test (Table 5). 

Precipitation had the largest contribution to variations in community characteristics, whereas soil properties had significant effect on the variations in leaf stoichiometric traits (Table 4 and Table 5).

## 4. Discussion

### 4.1. Community Characteristics and the Precipitation Gradient

Vegetation dynamics are tightly coupled with hydrological processes in arid and semi-arid ecosystems [2]. Previous studies have reported that precipitation plays an important role in regulating plant community structure and composition, with consequent influences on ecosystem functioning and potential feedback [1,4,48]. Our results showed that precipitation was the major driving force for variation of community characteristics and supported our hypothesis. In this arid region, the majority (more than 75%) of precipitation falls in July and August, shrub plants could use surface rainfall for survival in summer [30,49], and precipitation might be the main water resource for shrub plants. In addition, herb plants increased the community coverage, foliage projective cover, and species richness in rainy season. Therefore, water limitation might explain why single shrub-dominated community existed in S1–S5, while herb plant appeared in S6 and S7 (Table A1 and Table A2). Community height was significantly and negatively related to precipitation in our results, which was contrary to results in an alpine wetland ecosystem [50]. Potential mechanisms for this result may be that plants with greater height use more soil water at deeper depths than plants of smaller individual in hyperarid regions [51].

Our results showed that species richness and aboveground biomass increased linearly along the precipitation gradient (Figure 2), however, the maximum species richness and aboveground biomass did not appear in S7 with highest precipitation (Table 1). Decreased species richness and aboveground biomass appeared to be caused largely by the dominant shrub plant (*Kalidium cuspidatum*) [52]. Because *Kalidium cuspidatum* is a typical salt-secreting halophytic shrub, highly saline habitats with salt crust can develop [52], and distribution and growth of other plants were limited, therefore, species richness was low. Our results showed that species richness was significantly positive correlated with GSWC10 and GSWC30 (Table A5), which was contrary to results obtained in a previous study in an alpine wetland ecosystem [50], but was consistent with the results in arid and semiarid regions [3,53]. This difference might stem from the relatively small effects of interspecific competition in arid regions, whereas high species density in humid environments leads to greater interspecific competition and decreased species diversity [53]. These findings suggest that there is a positive interaction effect for plant diversity and upper soil water content in arid regions [54]. Although our results were consistent with previous conclusions obtained in arid and semi-arid regions, our results were derived from one-time-filed observations; the relationship between plant communities and precipitation may change in different seasons, long-term study are necessary in future.

### 4.2. Leaf Stoichiometric Traits and the Precipitation Gradient 

Leaf stoichiometric traits along a natural precipitation gradient in an arid desert habitat might be different from those reported at regional scales [35,55,56]. Our results showed that the mean of leaf C was 301.22 mg g^−1^, which was significantly lower than in other arid regions and lower than the average of global flora [21,25,55,56]. This difference might be because drought and salt stress inhibit desert plant photosynthesis by reducing stomatal conductance and water potential, and drought and salt stress lead to increased metabolic costs and decreasing C fixation [55,57]. The mean leaf N was 18.81 mg g^−1^, significantly lower than that in other arid regions [35,55,56], but nearly equivalent with Chinese flora and global flora [20,21,25]. Killingbeck et al. [58] reported that the average of leaf N was 22.0 mg g^−1^ based on 78 species of desert plant leaves. In our study, the fact that the leaf N of dominant species tended to be relatively low was largely due to the lower soil N and a relative lack of symbiotic N fixer [35]. 

Previous studies have reported that P is considered the major growth-constraining nutrients in plant communities in China compared with the global average [20]. However, some studies reported that soil P content have large variation across China and show an increasing trend from humid region to arid region [59]. In our study, the mean leaf P was 1.74 mg g^−1^, higher than that of the Loess Plateau and the average of Chinese flora [20,35], but nearly equivalent with that in other arid regions and the average of global flora [21,25,55,57]. Relative high leaf P content appeared to be caused largely by high soil P content, due to leaf P being tightly coupled with soil P [20]. An N/P ratio less than 14 indicates N constraint, while an N/P ratio more than 16 indicates P constraint. With an N/P ratio between 14 and 16, either or both N or P constrain plant growth [60]. In the present study, the leaf N/P ratio in this region was 12.7, indicating that plant growth was largely constrained by N. This is consistent with some previous studies in desert ecosystems [55,61], but differs from other studies in grassland and woodland [62,63]. This difference indicated that P might play an important role in plant distribution patterns in relative humid ecosystems in China. The relative deficiency in soil N content and the relatively adequate soil P content could possibly explain why N content is the key limiting factor for the plant N/P pattern in desert ecosystems [61].

Some studies on leaf stoichiometric traits-climate-soil relationships have been carried out at local, regional, or global scales providing further understanding of the mechanisms of vegetation dynamics in response to global climate change [20,25,55,64]. Reich and Oleksyn et al. [25] observed that leaf N, leaf P and N/P ratio were significantly related to latitude and mean annual temperature at a global scale. However, Kerkhoff et al. [64] reported that leaf N and leaf P were not related to the latitude, but leaf N/P ratio significantly decreased with increased latitude based on 1054 worldwide plant species. Han et al. [20] reported that leaf N and P of 753 plant species in China were significantly related with latitude and mean annual temperature, but leaf N/P ratio was not related to latitude. This discrepancy may be attributed to the different nutrient limitations in the different regions [28]. Our results showed that there was no significant linear relationship between leaf C/N/P stoichiometry and precipitation at the regional scale (Figure 3), and further proved leaf stoichiometric traits of different plant functional groups fluctuated significantly and the climate varied relatively little at a regional scale, so that variations of leaf stoichiometric traits modulated by the climate are non-significant [35]. Our results showed that leaf K was significantly and negatively related to precipitation (Figure 3), similar results were reported that ability of resisting drought and absorbing water for *Erica multiflora* L. depend on obtaining more K element in arid environment [65]. Our sampling captured a relatively narrow range of precipitation, given the complex relationships between precipitation and vegetation, detail field investigations in different seasons at large scale in arid region should be conducted to elucidate the responses of different functional groups or community-level leaf stoichiometric traits to precipitation in next research.

### 4.3. Soil Properties and the Precipitation Gradient

Soil properties play important roles in regulating plant community structure and composition in arid and semi-arid ecosystem, especially the non-phreatophyte species [1]. Our result showed that upper soil water content (0–30 cm) showed a significantly increasing trend with increasing precipitation (Figure 4), and was consistent with results obtained in arid and semi-arid region [3,7]. In our study, with increasing rainfall and decreasing evaporation in summer, soil moisture at soil surface from S1 to S7 increased. Some studies reported that soil with high bulk density has low water holding capacity in the surface soil and might induce drought stress in the surface soil in arid regions [66,67]. However, soil bulk density in our study did not show a significant decrease with increasing precipitation, and soil pH remained relatively constant with increasing precipitation (Figure 4). These results appeared to be caused by soil parent material that mainly composes of sand [30]. Thomey et al. [68] and Noy-Meir [4] found that large rainfall events result in a significantly high pulse-response in the upper soil water content (0–16 cm) in arid regions, and deep soil water content change controlled by precipitation are non-significant due to relative small precipitation and high evaporation. Our results also showed that deeper soil water content (30–50 cm) did not show a significant increase with increasing precipitation. However, the largest soil water content appeared in S1 (Figure 4). In addition, high soil electrical conductivity in S1 indicated that the soil water content (30–50 cm) may be affected by groundwater.

Previous studies have reported that precipitation can directly and indirectly affect soil properties via improved plant-soil feedback responses [1,48]. Zhou et al. [7] reported that precipitation may regulate plant production and decomposition and then affect soil C and soil N. Some studies have observed that soil C and N increased with increasing precipitation [69,70], and this was consistent with our results that soil total N and total C significantly increased with precipitation (Figure 4). The loss of soil water would increase organic matter decomposition rates and affect net N mineralization in hyperarid regions, which leads to losses of soil C and N content [71]. 

## 5. Conclusions

This study comprehensively characterized plant community characteristics, leaf stoichiometric traits, and soil properties along a precipitation gradient in an arid area of China. The analysis indicated that precipitation had a positive effect on species richness, aboveground biomass, community coverage, FPC, and LAI, but it significantly decreased community height, and precipitation was an important factor that affected soil properties, including soil water and soil nutrition. Whereas, soil properties, rather than precipitation, were drivers of desert plant leaf stoichiometric traits. The growth of desert plants might be more limited by N rather than P in this region. Given the different roles of precipitation and soil properties in leaf stoichiometric traits and community characteristics, these environmental factors should be involved in biogeochemical simulation models and degraded ecosystem restoration in arid areas. 

## Figures and Tables

**Table 1:** Characteristics of plant community and mean annual rainfall in the Heihe River Basin. Values are means ± SD.

Site	Locations	Annual Rainfall (mm)	Altitude (m)	Dominant Species	Species Richness	Aboveground Biomass (g/m^2^)	Coverage (%)	Height (cm)	Foliage Projected Cover (%)	Leaf Area Index
S1	42°16.13′ N 101°22.46′ E	35	920	*Haloxylon ammodendron*	1	101.8 ± 56.6	5.7 ± 0.5	152 ± 48.7	7.2 ± 3.5	0.8 ± 0.1
S2	42°30.87′ N 101°15.07′ E	39	982	*Nitraria sibirica* Pall.	1	60.3 ± 31.4	7.3 ± 2.0	25.4 ± 3.7	8.9 ± 2.5	0.1 ± 0.0
S3	40°16.90′ N 98°41.03′ E	69	1227	*Nitraria praevisa* Bobr.	2	72.7 ± 23.7	16.0 ± 6.0	26.6 ± 1.1	15.6 ± 2.5	0.2 ± 0.0
S4	39°56.53′ N 98°59.91′ E	82	1326	*Artemisia desertorum*	3	77.6 ± 23.5	17.6 ± 5.0	62.1 ± 14.1	12.2 ± 3.5	0.4 ± 0.1
S5	39°56.53′ N 100°46.17′ E	149	1655	*Kalidium gracile*	3	230.0 ± 126.6	19.1 ± 0.1	17.5 ± 1.5	27.8 ± 2.5	0.6 ± 0.1
S6	38°49.09′ N 100°59.24′ E	162	1714	*Salsola passerina*	5	316.0 ± 69.0	22.2 ± 3.3	27.9 ± 4.3	24.4 ± 2.5	0.8 ± 0.1
S7	38°37.55′ N 101°5.25′ E	209	2016	*Kalidium cuspidatum*	3	234.8 ± 45.1	27.3 ± 5.5	14.2 ± 0.3	37.8 ± 5.1	0.9 ± 0.1

**Table 2:** Leaf stoichiometric traits for dominant species along a precipitation gradient in the Heihe River Basin.

Site	C (mg/g)	N (mg/g)	P (mg/g)	K (mg/g)	C/N	C/P	N/P
S1	334.63 ± 11.39	21.06 ± 0.74	1.15 ± 0.01	20.29 ± 0.23	15.89 ± 0.03	291.03 ± 11.05	18.32 ± 0.73
S2	193.69 ± 3.34	13.49 ± 0.21	1.34 ± 0.01	15.68 ± 0.09	14.35 ± 0.12	144.63 ± 2.52	10.08 ± 0.18
S3	351.28 ± 28.75	26.74 ± 1.44	2.55 ± 0.11	18.43 ± 1.14	13.12 ± 0.19	138.18 ± 13.06	10.52 ± 0.71
S4	467.27 ± 2.02	22.83 ± 0.45	2.84 ± 0.04	29.04 ± 0.43	20.48 ± 0.19	164.78 ± 3.00	8.05 ± 0.09
S5	176.45 ± 2.43	12.86 ± 0.28	1.63 ± 0.03	12.82 ± 0.48	13.72 ± 0.11	108.19 ± 3.51	7.89 ± 0.32
S6	238.86 ± 3.67	16.46 ± 0.45	1.83 ± 0.08	18.16 ± 0.92	14.51 ± 0.17	130.40 ± 7.49	8.99 ± 0.62
S7	346.35 ± 27.43	18.19 ± 1.61	0.82 ± 0.05	8.70 ± 0.52	19.05 ± 0.24	420.55 ± 9.83	22.08 ± 0.80
Mean	301.22 ± 99.05	18.81 ± 4.86	1.74 ± 0.70	17.59 ± 6.08	15.88 ± 2.68	199.68 ± 108.61	12.27 ± 5.34

**Table 3:** Soil properties in different sites along a precipitation gradient in the Heihe River Basin. Values are means ± SD. Abbreviations: GSWC10, soil water content (0–10 cm); GSWC30, soil water content (10–30 cm); GSWC50, soil water content (30–50 cm); SBD, soil bulk density; TN, soil total nitrogen; TC, soil total carbon; C/N, soil C/N ratio; AP, soil available phosphorus, AK, soil available potassium content; pH, soil pH; EC, soil electrical conductivity.

Sites	GSWC10 (%)	GSWC30 (%)	GSWC50 (%)	SBD (g/cm^−3^)	TN (mg/g)	TC (mg/g)	C/N	AP (mg/kg)	AK (mg/kg)	pH	EC (ms/cm)
S1	1.10 ± 0.56	6.02 ± 2.22	11.38 ± 0.59	1.36 ± 0.09	0.45 ± 0.05	12.98 ± 4.65	29.22 ± 11.06	3.82 ± 1.23	312.36 ± 74.79	8.38 ± 0.22	6.51 ± 1.47
S2	0.22 ± 0.09	0.84 ± 0.18	1.53 ± 0.48	1.72 ± 0.09	0.57 ± 0.04	4.05 ± 0.99	7.18 ± 2.18	2.91 ± 0.63	118.28 ± 34.89	8.89 ± 0.36	0.46 ± 0.15
S3	1.41 ± 0.85	4.23 ± 2.74	5.57 ± 4.06	1.27 ± 0.19	0.88 ± 0.45	16.10 ± 1.62	21.91 ± 11.32	10.16 ± 4.23	237.94 ± 106.37	8.52 ± 0.12	6.85 ± 2.14
S4	0.42 ± 0.26	1.60 ± 0.57	2.47 ± 0.29	1.58 ± 0.04	0.39 ± 0.02	12.39 ± 1.08	31.69 ± 2.93	2.37 ± 0.37	183.90 ± 17.84	8.89 ± 0.76	1.72 ± 0.96
S5	0.89 ± 0.34	4.22 ± 0.82	5.02 ± 0.40	1.25 ± 0.04	0.83 ± 0.12	17.69 ± 0.74	21.64 ± 2.34	5.00 ± 1.52	136.08 ± 47.98	8.71 ± 0.04	2.14 ± 0.24
S6	2.24 ± 0.82	2.86 ± 0.73	3.79 ± 0.39	1.49 ± 0.01	0.82 ± 0.08	21.99 ± 1.43	26.73 ± 0.96	3.60 ± 0.60	68.40 ± 15.29	8.70 ± 0.16	1.26 ± 0.21
S7	3.33 ± 0.60	8.69 ± 1.22	10.42 ± 0.96	1.54 ± 0.11	0.99 ± 0.77	23.31 ± 1.18	23.62 ± 0.55	3.16 ± 0.44	175.05 ± 19.84	8.59 ± 0.07	3.41 ± 0.06

**Table 4:** Marginal and conditional effects obtained from the forward selection of the Monte Carlo test for community characteristics. The abbreviations are same as Table 3.

Marginal Effects		Conditional Effects		*p* Value	*F* Value
Environmental Variables	Eigenvalues	Environmental Variables	Eigenvalues		
Precipitation	60.5	Precipitation	60.5	0.001	29.1
Soil total carbon	42.5	Soil C/N	12.8	0.001	8.6
Soil total nitrogen	33.2	GSWC50	5.1	0.002	4.5
GSWC10	25.1	Soil total nitrogen	3.9	0.004	5.7
Soil available K	18.8	Soil available P	3.8	0.034	2.8
Soil C/N	11.7	SEC	2.2	0.082	2.2
GSWC30	9.3	Soil bulk density	1.7	0.190	1.6
SEC	6.3	Soil total carbon	1.5	0.193	1.6
GSWC50	4.3	GSWC10	1.2	0.115	2.0
Soil bulk density	3.4	GSWC30	1.1	0.395	1.0
Soil available P	1.8	Soil available K	0.7	0.391	1.0
Soil pH	1.6	Soil pH	0.6	0.460	0.9

**Table 5:** Marginal and conditional effects obtained from the forward selection of the Monte Carlo test for leaf stoichiometric traits. The abbreviations are same as Table 3.

Marginal Effects		Conditional Effects		*p* Value	*F* Value
Environmental Variables	Eigenvalues	Environmental Variables	Eigenvalues		
GSWC50	34.6	GSWC50	34.6	0.001	10.1
GSWC30	31.6	Soil C/N	11.7	0.002	7.4
GSWC10	21.9	Soil bulk density	16.0	0.003	5.8
Soil available K	14.7	GSWC30	5.9	0.007	4.5
SEC	12.9	SEC	12.4	0.010	5.7
Precipitation	10.1	Soil available K	3.6	0.038	3.5
Soil C/N	9.8	Soil total carbon	2.6	0.064	3.0
Soil total nitrogen	7.4	Precipitation	2.4	0.137	2.0
Soil total carbon	6.1	Soil available P	1.2	0.320	1.2
Soil available P	5.7	GSWC10	1.0	0.376	1.0
Soil pH	5.3	Soil total nitrogen	0.9	0.449	0.8
Soil bulk density	2.3	Soil pH	0.4	0.658	0.5

## Appendix A

**Table A1:** List of plant species in the seven sampling sites.

Sampling Sites	Family	Species
S1 (1 species including 1 shrub)	Chenopodiaceae	*Haloxylon ammodendron* (C. A. Mey.) Bunge
S2 (1 species including 1 shrub)	Zygophyllaceae	*Nitraria sibirica* Pall.
S3 (2 species including 2 shrubs)	Zygophyllaceae	*Nitraria praevisa* Bobr.
	Solanaceae	*Lycium ruthenicum* Murr.
S4 (3 species including 3 shrubs)	Asteraceae	*Artemisia desertorum* Spreng.
	Ephedraceae	*Ephedra przewalskii* Stapf.
	Polygonaceae	*Calligonum mongolicum* Turcz.
S5 (3 species including 3 shrubs)	Chenopodiaceae	*Kalidium gracile* Fenzl
	Chenopodiaceae	*Salsola passerina* Bunge
	Chenopodiaceae	*Sympegma regelii* Bunge
S6 (7 species including 5 shrubs and 2 herbages)	Chenopodiaceae	*Kalidium gracile* Fenzl
	Chenopodiaceae	*Salsola passerina* Bunge
	Chenopodiaceae	*Sympegma regelii* Bunge
	Leguminosae	*Caragana roborovskyi* Kom.
	Zygophyllaceae	*Nitraria roborowskii* Kom.
	Chenopodiaceae	*Agriophyllum squarrosum* (L.) Moq.
	Chenopodiaceae	*Halogeton glomeratus* (Bieb.) C. A. Mey.
S7 (4 species including 3 shrubs and 1 herbage)	Chenopodiaceae	*Kalidium cuspidatum* (Ung. Sternb.) Grub.
	Chenopodiaceae	*Salsola passerina* Bunge
	Tamaricaceae	*Reaumuria songarica* (Pall.) Maxim.
	Zygophyllaceae	*Zygophyllum fabago* L.

**Table A2:** Information of sampling sites. Shrub layer: HA, *Haloxylon ammodendron*; NS, *Nitraria sibirica*; NP, *Nitraria praevisa*; AD, *Artemisia desertorum*; KG, *Kalidium gracile*; SP, *Salsola passerina*; SR, *Sympegma regelii*; KC, *Kalidium cuspidatum*. Herb layer: AS, *Agriophyllum squarrosum*; HG, *Halogeton glomeratus*; ZF, *Zygophyllum fabago*.

Site	Annual Rainfall (mm)	Important Value of Major Species in Shrub Layer								Important Value of Major Species in Herb Layer		
		HA	NS	NP	AD	KG	SP	SR	KC	AS	HG	ZF
S1	35	1.00										
S2	39		1.00									
S3	62			0.76								
S4	82				0.72							
S5	149					0.51	0.31					
S6	162						0.61	0.22		0.85	0.15	
S7	209						0.26		0.69			1

**Table A3:** One-way ANOVA of community characteristics and leaf stoichiometric traits among the sampling sites across the middle and lower reaches of Heihe River Basin. *** indicates significant difference at *p* < 0.001.

Community Characteristics/Leaf Stoichiometric Traits	Sum of Squares	df	Mean Square	*F*	Sig.
Species richness	1.153	6	0.192	38.79	<0.001 ***
Aboveground biomass	1.601	6	0.267	8.75	<0.001 ***
Community coverage	1.148	6	0.191	18.51	<0.001 ***
Community height	2.187	6	0.364	62.05	<0.001 ***
Foliage projective cover	1.403	6	0.234	14.80	<0.001 ***
Leaf area index	2.127	6	0.354	76.44	<0.001 ***
Leaf C	0.430	6	0.072	175.76	<0.001 ***
Leaf N	0.246	6	0.041	109.19	<0.001 ***
Leaf P	0.651	6	0.108	456.43	<0.001 ***
Leaf K	0.487	6	0.081	253.59	<0.001 ***
Leaf C/N	0.098	6	0.016	319.26	<0.001 ***
Leaf C/P	0.815	6	0.136	306.01	<0.001 ***
Leaf N/P	0.564	6	0.094	241.23	<0.001 ***

**Table A4:** One-way ANOVA of soil properties among the sampling sites across the middle and lower reaches of Heihe River Basin. *, ** indicate significant difference at *p* < 0.05 and *p* < 0.01, respectively.

Soil Properties	Sum of Squares	df	Mean Square	*F*	Sig.
Gravimetric soil water content (0–10 cm)	2.596	6	0.433	3.24	<0.033 *
Gravimetric soil water content (10–30 cm)	4.144	6	0.691	12.62	<0.001 **
Gravimetric soil water content (30–50 cm)	3.862	6	0.644	18.01	<0.001 **
Soil bulk density	0.251	6	0.042	24.39	<0.001 **
Soil total nitrogen	2.034	6	0.339	22.26	<0.001 **
Soil total carbon	6.793	6	1.132	56.56	<0.001 **
Soil C/N	4.751	6	0.792	28.68	<0.001 **
Soil available P	3.665	6	0.611	23.76	<0.001 **
Soil available K	4.215	6	0.702	24.02	<0.001 **
Soil pH	0.007	6	0.001	2.330	<0.041 *
Soil electrical conductivity	19.360	6	3.227	25.36	<0.001 **

**Table A5:** Pearson’s correlation coefficients (r) among community characteristics, leaf stoichiometric traits, and environmental factors in different sites along a precipitation gradient in the Heihe River Basin. Significant correlations at *p* < 0.05 and *p* < 0.01 are shown in bold and in bold with underline, respectively. Abbreviations: SR, species richness; AGB, aboveground biomass; COVER, community coverage; Height, community height; FPC, foliage projective cover; LAI, leaf area index. Other abbreviations are described in Table 3.

Community Characteristics and Leaf Stoichiometric Traits	GSWC10	GSWC30	GSWC50	SBD	STN	STC	C/N	SAP	SAK	pH	EC
SR	**0.742**	**0.642**	0.370	−0.152	**0.752**	**0.700**	−0.040	0.179	−0.132	−0.144	0.100
AGB	**0.449**	0.342	0.130	−0.167	0.386	**0.730**	0.273	−0.155	−0.375	−0.044	−0.218
COVER	**0.537**	0.290	0.077	−0.059	**0.560**	**0.739**	0.193	0.017	−0.345	0.077	−0.158
HEIGHT	−0.248	0.189	0.414	−0.131	**−0.539**	−0.177	**0.469**	−0.179	**0.628**	−0.249	**0.477**
FPC	**0.678**	**0.463**	0.226	−0.149	**0.698**	**0.752**	0.003	−0.046	−0.359	−0.136	−0.157
LAI	**0.573**	**0.581**	**0.674**	−0.196	0.103	**0.623**	**0.496**	−0.285	0.112	−0.232	0.133
Leaf C	0.067	0.122	0.170	0.071	−0.242	0.075	**0.517**	−0.033	0.429	−0.069	0.333
Leaf N	0.037	0.090	0.180	−0.245	−0.070	0.064	0.383	0.416	**0.534**	−0.242	**0.649**
Leaf P	−0.407	**−0.518**	**−0.548**	−0.126	−0.191	−0.122	0.243	0.315	−0.011	0.159	0.027
Leaf K	**−0.541**	**−0.524**	−0.357	0.122	**−0.607**	−0.366	0.375	−0.096	0.200	0.110	0.022
Leaf C/N	0.171	0.173	0.148	0.431	−0.257	0.114	0.413	**−0.538**	0.104	0.147	−0.170
Leaf C/P	**0.596**	**0.724**	**0.746**	0.143	0.145	0.326	0.174	−0.257	0.340	−0.246	0.288
Leaf N/P	**0.583**	**0.748**	**0.813**	0.035	0.172	0.286	0.104	−0.140	0.411	−0.335	0.413
